# Pulsed Field Ablation for Atrial Fibrillation in Patients With Obesity: Procedural and Clinical Outcomes

**DOI:** 10.1111/pace.70145

**Published:** 2026-01-26

**Authors:** Francis J. Ha, Duron Prinsloo, Edwin Xu, Hui Chen Han, Nitesh Nerlekar, Adam J. Brown, Emily Kotschet

**Affiliations:** ^1^ Victorian Heart Institute and Victorian Heart Hospital Monash University Clayton Victoria Australia

**Keywords:** atrial fibrillation, pulsed field ablation, obesity

## Abstract

**Background:**

Procedural and clinical outcomes in patients with obesity undergoing catheter ablation for atrial fibrillation (AF) have been mixed. Clinical data in the context of pulsed field ablation (PFA) are currently limited in this patient cohort.

**Methods:**

We conducted an observational study of consecutive patients undergoing PFA with a pentaspline catheter (Farapulse^TM^, Boston Scientific) for AF at a tertiary center comparing procedural and clinical outcomes in patients with body mass index (BMI) <30 kg/m^2^ (nonobese), 30–34.9 kg/m^2^ (obese), and ≥35 kg/m^2^ (morbidly obese). Baseline and procedural characteristics, procedural outcomes (major and minor complications) and clinical outcomes were collected. Arrhythmia recurrence was defined as AF, atrial flutter or atrial tachycardia >30 s at any follow‐up.

**Results:**

537 (95%) out of 564 consecutive patients underwent de novo PFA for AF between 2022 and 2025 with a recorded BMI; 326 (60.7%) were nonobese, 117 (21.8%) were obese and 94 (17.5%) morbidly obese patients. Proportion of females increased according to BMI (*p* = 0.003). Obesity correlated with CHA_2_DS_2_‐VASc score, hypertension, diabetes mellitus, obstructive sleep apnea and heart failure with reduced ejection fraction (*p* < 0.05 for all). Persistent AF was more common (*p* = 0.048). Radiation exposure was significantly increased in obese patients (*p* < 0.001). There was no difference in major (1.2%–3.2%) or minor (11%–15%) procedural complications between groups. There was no difference in freedom from arrhythmia recurrence between groups (67%–70% at median follow‐up 5.9–7.4 months).

**Conclusion:**

Patients with obesity had no signal of increased risk detected with regard to procedural safety and clinical outcomes with PFA for AF compared with nonobese patients. These findings apply to a specific pentaspline PFA catheter rather than PFA technology in general.

## 1 Introduction

Atrial fibrillation (AF) is the most common sustained arrhythmia and a major contributor to cardiovascular morbidity and healthcare burden worldwide [1]. Guidelines recommend catheter ablation as a cornerstone of rhythm‐control therapy for symptomatic AF [2]. Conventional thermal approaches to ablation, such as radiofrequency and cryoablation, are effective but nonselective and carry the risk of collateral injury to adjacent structures, such as the oesophagus, pulmonary veins, and phrenic nerve [3, 4]. Pulsed field ablation (PFA) has emerged as a newly adopted modality that achieves ablation through irreversible electroporation with greater tissue selectivity, the potential to reduce collateral injury and improved procedural workflow.

Obesity is a major driver for risk of atrial fibrillation (AF). For each 5 kg/m^2^ increase in body mass index (BMI), there is up to a 29% increased risk of AF both in the broader population and after AF ablation [6]. Catheter ablation in patients with obesity has been limited by concern for increased arrhythmic recurrence, increased risk of procedural complications and resource limitations [7, 8]. However, the PRAGUE‐25 trial showed that catheter ablation in patients with paroxysmal or persistent AF and BMI of 30–45 kg/m^2^ was associated with a significantly greater freedom from AF at 12 months compared with lifestyle modification and anti‐arrhythmic drug therapy (73% compared with 35%, respectively) [9].

With the advent of PFA for AF, real‐world clinical outcomes in patients with obesity are currently lacking. In this study, we evaluated procedural safety and clinical outcomes of patients with obesity undergoing PFA for AF using a pentaspline catheter system compared with nonobese patients.

## 2 Methods

We performed a retrospective analysis of consecutive patients undergoing PFA with a pentaspline catheter (Farapulse, Boston Scientific, USA) for paroxysmal or persistent AF at a large tertiary center in Australia from November 2022 to June 2025. Consecutive patients undergoing de novo PFA were included. Patients were analyzed in three groups according to body mass index (BMI): <30 kg/m^2^ (nonobese), 30–34.9 kg/m^2^ (obese), and ≥35 kg/m^2^ (morbidly obese). Approval for this study was granted by the Monash Health Human Research Ethics Committee.

### 2.1 Baseline Demographics

Baseline demographic and clinical variables were obtained from electronic medical records, including age, sex, body mass index (BMI), AF phenotype (paroxysmal or persistent), and relevant comorbidities. Obstructive sleep apnea was recorded where documented on patient known history. For each patient we calculated the CHA_2_DS_2_‐VASc score and assessed renal function using the estimated glomerular filtration rate (eGFR). When available, echocardiographic indices—left ventricular ejection fraction (LVEF) and indexed left atrial volume (LAVI)—were recorded. Pre‐ablation pharmacotherapy (antiarrhythmic drugs and oral anticoagulants) was documented. Paroxysmal AF was defined as episodes resolving spontaneously within <7 days, and persistent AF as episodes lasting ≥7 days.

### 2.2 PFA Procedure

All patients underwent PFA using the Farapulse system under general anesthesia and intubation. Pulmonary venous anatomy and left atrial appendage (LAA) assessment were performed intra‐procedurally using transesophageal echocardiography (TOE) or contrast venography, according to physician discretion during the procedure. Peri‐procedural anticoagulation strategy followed individual physician preference.

Femoral venous access was obtained via 6Fr and 7Fr sheaths. Guidance with ultrasonography was physician dependent. In most patients, a multipolar catheter was positioned in the coronary sinus for intra‐procedural monitoring. Trans‐septal access was performed under TOE and hemodynamic monitoring guidance. Intravenous heparin was administered to maintain an activated clotting time (ACT) ≥350 s. Patients presenting in AF at the commencement of the procedure were cardioverted prior to ablation. Atropine was routinely administered to reduce the risk of vagal response.

A pentaspline catheter (31 mm or 35 mm) was selected according to left atrial size. Each pulmonary vein was treated using both “flower” and “basket” configurations at 2 kV, with a minimum of six paired applications per vein delivered over a Rosen wire (Cook Medical, US). Posterior wall isolation and ablation of non‐pulmonary vein targets were undertaken at the discretion of the operator. While the number of applications to the posterior wall was at operator discretion, the typical number of applications to the superior vena cava was two paired applications. From February 2025 onwards, electroanatomic mapping was used concurrently through the Faraview software (Boston Scientific, US) integrated into the Farapulse system.

Following completion of ablation, all catheters were withdrawn, and femoral access sites were closed with either a figure‐of‐eight suture or a vascular closure device such as the Perclose ProStyle system (Abbott Vascular, USA) according to operator preference. Patients were monitored in the immediate recovery area, with same‐day discharge performed in accordance with institutional protocols, and inpatient admission arranged when clinically indicated or at the proceduralist's discretion.

Procedural data collected included lesion set type (e.g., pulmonary vein isolation, posterior wall isolation, superior vena cava isolation), total number of PFA applications, overall procedure time, left atrial catheter dwell time, PFA application time (from first to final application), fluoroscopy duration, and radiation exposure quantified as air kerma (mGy) and dose‐area product (µGy·m^2^).

### 2.3 Clinical Outcomes

Procedural complications were classified as major or minor. Major complications were defined as death, atrio‐oesophageal fistula, symptomatic pulmonary vein stenosis, cardiac tamponade, stroke, coronary artery spasm, persistent phrenic nerve injury, major vascular complication requiring surgical intervention, or new dialysis requirement. Minor complications included systemic air embolism, transient ischemic attack, transient phrenic nerve injury, minor vascular complications requiring nonsurgical intervention (e.g., FemoStop application or additional manual compression), pericarditis, pulmonary oedema, hemolysis, and transient renal impairment (defined as a doubling of serum creatinine compared with baseline). For patients admitted following the procedure, renal function on the first post‐procedural day was recorded when available.

Follow‐up consisted of a standardized telephone assessment by nursing staff at seven days post‐procedure. Thereafter, patients were reviewed either in the tertiary center arrhythmia clinic or by their private cardiologist. Rhythm monitoring was performed according to clinical indication and symptom presentation. Arrhythmia recurrence was defined as any symptomatic AF, atrial flutter, atrial tachycardia or documented arrhythmia lasting more than 30 s at any follow‐up. No blanking period was applied. Detection methods included 12‐lead electrocardiography for sustained arrhythmia, ambulatory Holter monitoring, implantable device check, or validated wearable monitors. Data were collected regarding subsequent electrical cardioversion, unplanned hospital readmission, and the requirement for repeat catheter ablation.

### 2.4 Statistical Analysis

Categorical variables are reported as frequencies with corresponding percentages, while continuous variables are expressed as mean with standard deviation (SD) or median with interquartile range (IQR), according to data distribution. Between‐group comparisons were performed using Pearson's chi‐square test for categorical variables and Student's *t*‐test or one‐way analysis of variance (ANOVA) for continuous variables, as appropriate. Logistic regression was performed to identify potential risk factors for major procedural complications and reported as odds ratio (OR) with 95% confidence interval (CI). Freedom from arrhythmia recurrence was assessed using Kaplan–Meier survival analysis, with comparisons between groups performed using the log‐rank test. Cox proportional hazards regression was performed on potential risk factors and risk of arrhythmia recurrence and reported as hazard ratio with 95% CI. Covariates included in multivariate analysis were based on known potential associations with clinical outcomes from previously published studies including CHA_2_DS_2_‐VASc score and its individual components and type of AF [10]. Two‐sided *p*‐value of <0.05 was considered statistically significant. Statistical analyses were conducted using Stata MP 14.0 (StataCorp LP, College Station, TX, USA).

## 3 Results

### 3.1 Baseline Demographics

A total of 564 consecutive patients underwent de novo PFA for AF during the study period, of which 537 patients (95%) had a recorded BMI. This included 326 (60.7%) nonobese patients (BMI <30 kg/m^2^), 117 (21.8%) obese patients (BMI 30–34.9 kg/m^2^) and 94 (17.5%) morbidly obese patients (BMI ≥35 kg/m^2^; Table 1). Median age was similar across groups (63–64 years; *p* = 0.83). The proportion of females increased according to BMI cohort (30% in the nonobese cohort, 39% in the obese cohort, 48% in morbidly obese cohort; *p* = 0.003). Median BMI in the nonobese cohort was 26.5 kg/m^2^ (IQR 24.5–28.4), 32.0 kg/m^2^ (IQR 31.0–33.6) and 37.2 kg/m^2^ (IQR 36.2–39.5) in the morbidly obese group (*p* < 0.001). CHA_2_DS_2_‐VASc score was higher with increasing BMI (*p* = 0.003). Hypertension (*p* < 0.001), obstructive sleep apnea (*p* < 0.001) and heart failure with reduced ejection fraction (*p* < 0.019) were more common with increasing BMI. Diabetes mellitus was more common amongst obese and morbidly obese patients compared with nonobese patients (*p* = 0.01). Persistent AF was more common in obese and morbidly obese patients compared with nonobese patients (44% vs. 33%; *p* = 0.048). Indexed left atrial volume were similar between groups (*p* = 0.12).

**TABLE 1:** Baseline demographics.

	BMI <30 kg/m^2^ *n* = 326	BMI 30–35 kg/m^2^ *n* = 117	BMI ≥35 kg/m^2^ *n* = 94	*p*‐value
Age, years (IQR)	64 (56–70)	64 (55–69)	63 (57–69)	0.83
Female (%)	97 (30)	46 (39)	45 (48)	**0.003**
BMI, kg/m^2^ (IQR)	26.5 (24.5–28.4)	32.0 (31.0–33.6)	37.2 (36.2–39.5)	**<0.001**
CHA_2_DS_2_‐VASc	1.8 ± 1.4	2.1 ± 1.5	2.3 ± 1.3	**0.003**
Comorbidities (%)				
Hypertension	147 (45)	62 (53)	68 (72)	**<0.001**
Vascular disease	37 (11)	18 (16)	17 (18)	0.17
Heart failure	47 (15)	30 (26)	14 (15)	**0.019**
Diabetes mellitus	27 (8)	21 (18)	14 (15)	**0.01**
Stroke/TIA	15 (5)	5 (4)	7 (7)	0.50
Obstructive sleep apnea	32 (10)	24 (21)	32 (34)	**<0.001**
eGFR, mL/min/1.73m^2^	82 (68–90)	83 (70–90)	82 (65–90)	0.75
Type of AF (%)				
Paroxysmal	218 (67)	65 (56)	54 (58)	**0.048**
Persistent	108 (33)	55 (44)	40 (43)	
Prior AF ablation (%)	45 (14)	18 (15)	8 (9)	0.30
LVEF %, median (IQR)	60 (54–60)	55 (45–60)	56 (50–60)	**0.006**
LAVI, ml/m^2^	42 (37–52)	48 (41–54)	41 (35–50)	0.12
Antiarrhythmic drug				
None	96 (31)	21 (19)	30 (33)	**0.049**
Flecainide	87 (28)	27 (25)	15 (16)	
Sotalol	82 (26)	40 (37)	29 (32)	
Amiodarone	47 (15)	21 (19)	18 (20)	
Not recorded	6 (2)	2 (2)	2 (2)	
Anticoagulation prior				
None	22 (7)	6 (5)	3 (3)	0.62
Apixaban	231 (72)	83 (71)	72 (77)	
Rivaroxaban	61 (19)	22 (19)	14 (15)	
Dabigatran	6 (2)	3 (3)	3 (3)	
Warfarin	2 (1)	3 (3)	1 (1)	
Not recorded	1	0	1	

Abbreviations: AF, atrial fibrillation; BMI, Body mass index; IQR, interquartile range; LAVI, left atrial volume indexed; LVEF, left ventricular ejection fraction; TIA, transient ischaemic attack.

### 3.2 Procedural Characteristics

There were no differences in ablation lesion set and number of PFA applications between groups (Table 2). Similarly, there were no differences in procedural time or left atrial catheter dwell times. Radiation exposure increased significantly with BMI for both air kerma (*p* < 0.001) and dose–area product (*p* < 0.001) despite similar fluoroscopy time between groups (median 15–17 min across groups; *p* = 0.15). Faraview electroanatomic mapping was available and used in 11%–13% patients with no difference in use according to BMI groups (*p* = 0.83).

**TABLE 2:** Procedural characteristics.

	BMI <30 kg/m^2^ *n* = 326	BMI 30–35 kg/m^2^ *n* = 117	BMI ≥35 kg/m^2^ *n* = 94	*p*‐value
Ablation lesion set				
Pulmonary vein	326 (100)	116 (99)	94 (100)	0.17
Posterior wall	120 (37)	57 (49)	40 (43)	0.08
Superior vena cava	11 (3)	4 (3)	2 (2)	0.82
Number of PFA applications				
Total applications	57 (50–69)	60 (50–72)	62 (52–70)	0.21
Pulmonary vein applications	50 (46–56)	50 (48–54)	50 (48–54)	0.35
Posterior wall applications	20 (18–26)	22 (20–26)	22 (20–24)	0.31
Superior vena cava applications	4 (4–6)	4 (4–4)	5 (4–6)	0.62
Procedural time, minutes	63 (51–76)	64 (49–75)	64 (54–80)	0.34
Left atrial catheter dwell time, minutes	40 (31–51)	41 (30–51)	42 (32–51)	0.95
Application time, minutes	26 (19–34)	28 (20–35)	26 (20–34)	0.65
Fluoroscopy time, minutes	17 (13–23)	17 (13–22)	19 (14–25)	0.15
Radiation dose, Air Kerma mGy	113 (73–187)	184 (119–299)	260 (174–410)	**<0.001**
Radiation dose, dose‐area product µGym^2^	1276 (822–2015)	1919 (1302–3399)	2753 (1854–4585	**<0.001**

Abbreviation: PFA, pulsed field ablation.

### 3.3 Procedural Complications

Overall rate of major complications ranged from 1.2% to3.2% across groups (*p* = 0.43). There were two deaths in the entire cohort, both occurring in nonobese patients. The first death was in a 62 year old male with severe ischemic cardiomyopathy where the catheter inadvertently fell back across the interatrial septum and PFA was unintentionally applied in proximity to the sinoatrial node resulting in junctional bradyarrhythmia with cardiogenic shock. Despite subsequent insertion of a permanent pacemaker, his cardiogenic shock persisted with progressive multi‐organ failure and he died on day one post‐procedure. The second death was in a 70 year old female with severe LV dysfunction (LVEF 30%) who underwent an uncomplicated elective PFA for persistent AF with planned same‐day discharge. However, the next day she presented to an external hospital with splenic and renal infarcts and ischemic bowel requiring bowel resection and did not survive. There were no cases of atrio‐oesophageal fistula, symptomatic pulmonary vein stenosis, coronary artery spasm, or persistent phrenic nerve injury. Cardiac tamponade occurred in one obese patient and one morbidly obese patient (*p* = 0.21); both were drained percutaneously without complication. Stroke was not significantly different between groups (0.6%–1%; *p* = 0.89). Overall rate of minor complications was likewise comparable (11%–15%; *p* = 0.71), most commonly including minor vascular complications (7%–9%; *p* = 0.69) and pericarditis (1.5%–5%; *p* = 0.11). Procedural complications are detailed in Table 3. There were no specific major anesthesia‐related complications. A history of chronic heart failure (OR 5.0, 95% CI 1.24–20.6, *p* = 0.024) and worse LVEF (OR 0.95, 95% CI 0.90–0.99, *p* = 0.046) were associated with major adverse procedural complications on univariate analysis. BMI as a continuous variable was not significantly associated with major procedural complications (*p* = 0.26), nor was age (*p* = 0.11), female sex (*p* = 0.55), CHA_2_DS_2_‐VASc (*p* = 0.39), hypertension (*p* = 0.80), obstructive sleep apnea (*p* = 0.71) or type of AF (*p* = 0.79). On multivariate analysis, a history of chronic heart failure was no longer associated with risk of major procedural complications (*p* = 0.10) adjusted for age, BMI, female sex and CHA_2_DS_2_‐VASc.

**TABLE 3:** Procedural complications.

	BMI <30 kg/m^2^ *n* = 326	BMI 30–35 kg/m^2^ *n* = 117	BMI ≥35 kg/m^2^ *n* = 94	*p*‐value
Major procedural complications (%)	4 (1.2)	2 (1.7)	3 (3.2)	0.43
Death	2 (0.6)	0	0	0.52
Atrio‐oesophageal fistula	0	0	0	
Symptomatic pulmonary vein stenosis	0	0	0	
Cardiac tamponade	0	1 (0.85)	1 (1)	0.21
Stroke	2 (0.6)	1 (0.85)	1 (1)	0.89
Coronary artery spasm	0	0	0	
Persistent phrenic nerve injury	0	0	0	
Major vascular complication	2 (0.6)	0	1 (1)	0.58
Dialysis requirement	1 (0.3)	0	0	0.72
Minor procedural complications (%)	41 (13)	13 (11)	14 (15)	0.71
Systemic air embolism	0	0	0	
Transient ischaemic attack	1 (0.3)	1 (0.9)	1 (1)	0.61
Transient phrenic nerve injury	0	0	0	
Minor vascular complication	30 (9)	8 (7)	7 (7)	0.69
Pericarditis	5 (1.5)	3 (3)	5 (5)	0.11
Pulmonary oedema	2 (0.6)	2 (1.7)	0	0.32
Haemolysis	1 (0.3)	0	0	0.72
Transient renal impairment	3/32 (9)	2/9 (22)	2/4 (50)	0.09

### 3.4 Clinical Outcomes

**FIGURE 1:**
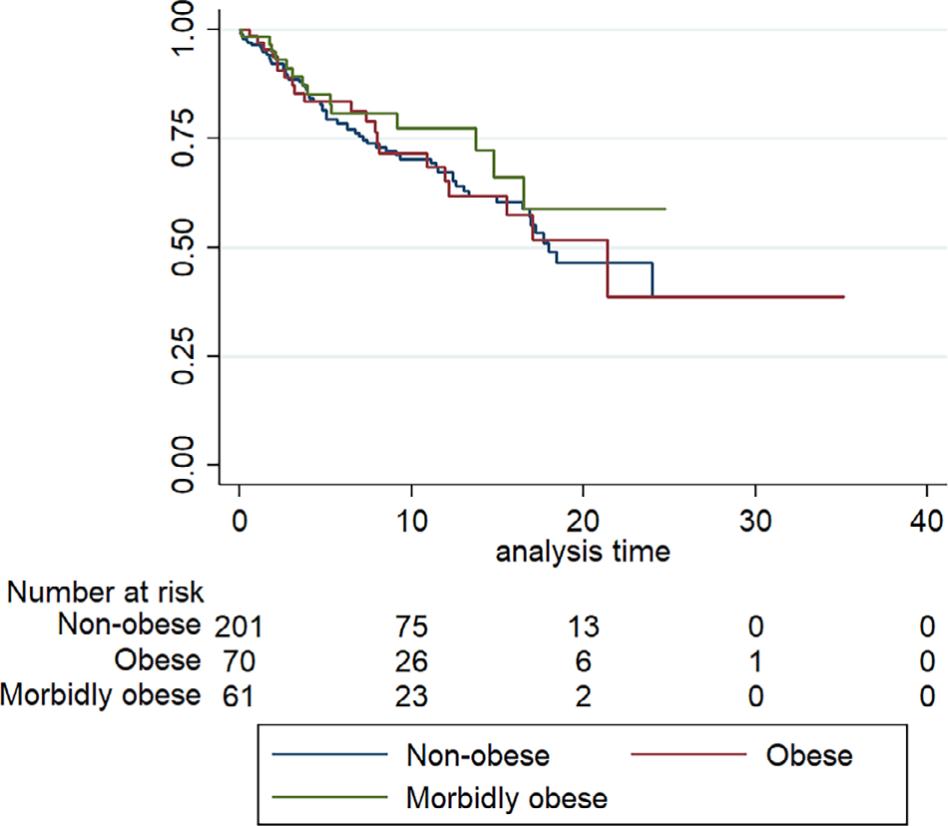
Freedom from arrhythmia recurrence between BMI groups.

There was no significant difference in freedom from arrhythmia recurrence between groups based on BMI category where follow‐up was available (*n* = 322; Figure 1). There was no difference in rate of follow‐up available between BMI groups (63% in the nonobese group, 62% in the obese group and 65% in the morbidly‐obese group; *p* = 0.88). Freedom from arrhythmia recurrence was 68% in the nonobese group (median follow‐up 5.9 months [IQR 2.6‐13.7]), 70% in the obese group (median follow‐up 6.1 months [IQR 2.2–12.7]) and 67% in the morbidly obese group (median follow‐up 7.4 months [IQR 2.7‐13.5]; *p* = 0.41 for difference) (Figure 1). There was no significant difference between rate of recurrence depending on type of AF (paroxysmal vs. persistent) in each group (nonobese patients, *p* = 0.71; obese patients, *p* = 0.44; morbidly‐obese patients, *p* = 0.97). On univariate Cox regression analysis, BMI as a continuous variable was not a significant predictor of arrhythmia recurrence (*p* = 0.86). Age (*p* = 0.56), female sex (*p* = 0.58), CHA_2_DS_2_‐VASc (*p* = 0.52), diabetes mellitus (*p* = 0.82), hypertension (*p* = 0.96), chronic heart failure (*p* = 0.87), obstructive sleep apnea (*p* = 0.79), LVEF (*p* = 0.86) and persistent AF (*p* = 0.88) similarly did not predict arrhythmia recurrence on univariate analysis. Subsequent multivariate analysis was not performed due to lack of statistical significance of any individual potential risk factor on univariate analysis. There was no difference between BMI groups for need for cardioversion (5%–6%; *p* = 0.90), hospitalization related to arrhythmia (overall 2.8%; *p* = 0.29) or redo‐ablation procedure (6.3%; *p* = 0.83). Ten patients had an implanted cardiac device with a median duration of arrhythmia recurrence follow‐up of 12.2 months (IQR 3.9–12.7 months). The limited number of patients with implanted cardiac device limited comparison of arrhythmia recurrence.

## 4 Discussion

We conducted an observational study of patients undergoing de novo PFA for AF at our tertiary center comparing procedural and clinical outcomes between obese and nonobese patients. We found that:

1. Patients with obesity were more likely female, and had higher prevalence of cardiovascular comorbidities including hypertension, diabetes mellitus, obstructive sleep apnea.
2. Patients with increasing obesity were more likely to have persistent AF at time of referral for catheter ablation.
3. Patients with obesity had increased radiation exposure however there was no difference in procedural safety with regard to major and minor procedural complications.
4. Clinical outcomes at follow‐up were similar across groups, with no significant differences in arrhythmia recurrence, need for cardioversion, repeat hospitalization, or redo ablation procedures.

Obesity is recognized to affect about 650 million adults and 340 million children worldwide [11]. The health consequences are well recognized and once again confirmed in our study with the increased proportion of cardiovascular comorbidities in patients with obesity [12]. In general, females are more obese than males and given the same BMI, women have a 10% higher fat mass compared with men although this may not necessarily confer a higher cardiometabolic risk [13, 14]. Increased weight is associated with atrial electroanatomic remodeling including increased left atrial volume, fibrosis, reduced conduction velocity and subsequently greater predisposition to inducible and spontaneous AF [15]. Thus it is unsurprising that a greater proportion of patients with obesity referred for catheter ablation had persistent AF compared with nonobese patients and our findings are similar [16]. Obstructive sleep apnea, while frequently coexists with obesity, itself confers an association with AF, [17] and when treated with CPAP results in reversal of atrial remodeling including faster conduction velocity and higher voltages in the left atrium [18].

It has been previously recognized that patients with obesity are exposed to greater cumulative radiation dose compared with normal‐weight patients in relation AF ablation and pre‐procedural planning [19, 20]. Obese patients receive a dose 75% higher than normal‐weight patients although most of this is related to preprocedural computed tomography rather than fluoroscopy during the AF ablation procedure itself [19]. At our center, we do not routinely perform a pre‐procedural computed tomography for AF ablation due to peri‐procedural trans‐oesophageal imaging guidance and resource limitation. Nevertheless, we similarly observed marked increases in radiation dose exposure in obese patients relative to nonobese counterparts. This effect should not be discounted as such medical radiation exposure is cumulative and radiation‐related lifetime cancer risk is increased in patients with obesity [21].

The impact of obesity in procedural safety and complications related to AF ablation is mixed. Registry data from the United States of 5841 patients undergoing AF ablation showed a risk of major procedural complications of 1.1%–1.7% across all subgroups of BMI with no significant difference between groups [22]. Conversely, in a propensity matched analysis of more recent data concerning 83,767 patients undergoing catheter ablation for AF in the United States, morbid obesity was associated with increased post‐procedural bleeding, acute kidney injury, respiratory complications and higher length of stay [23]. Similarly, a large US dataset of 106,462 AF ablations showed a slightly increased risk of in‐hospital complications in obese patients [24]. These studies evaluated thermal ablation and did not include PFA. Our study showed no statistically significant difference in rate of major procedural complications, although numerically patients with morbid obesity had 3.2% rate of major procedural complications amongst 94 patients. Similarly, there was no difference in minor procedural complications between groups with importantly no difference with regards to minor vascular access complications. The rate of pericarditis diagnosis ranged from 1.5%–5% in this cohort. This is possibly influenced by potential overdiagnosis from the emergency department after patients may re‐present with nonspecific chest pain and elevated troponin soon after PFA and empirically treated with anti‐inflammatories. Nevertheless, pericarditis has been reported with PFA despite its mechanism being predominantly nonthermal and should monitored carefully [25, 26]. Minor vascular access complications ranged from 7% to 9% which likely reflects how it was defined, including any further manual compression or requirement of a FemoStop device, as well as potential use of vascular ultrasound. A randomized trial has shown that the use of percutaneous vascular closure device reduces minor bleeding and ambulation time compared with conventional figure‐of‐eight suture [27]. However routine uptake of this approach may be limited by financial constraints.

Presence of obesity did not appear to affect procedural or left atrial catheter dwell time and with no difference in total fluoroscopy time either. These data are at least reassuring in the context of PFA and could reflect the reduced risk of AF ablation with this technology in general such as procedural times of about one hour, which extends to patients with obesity as well.

The lack of significant differences in arrhythmia outcomes between different BMI groups in our study is notable. Morbid obesity tends to reduce AF ablation success [16, 22, 28]. Left atrial epicardial adipose tissue, which is strongly associated with obesity, is a well‐established predictor of AF recurrence including after catheter ablation [29]. Outcomes from AF ablation may be improved if bariatric surgery is performed prior, which highlights the definitive role of weight loss as part of cornerstone treatment still [30]. Yet, randomized controlled data have shown that patients with obesity still derive marked benefit with catheter ablation over lifestyle modification and anti‐arrhythmic drug therapy [9]. While obese patients in the conservative treatment in this trial only achieved 5.5% weight loss compared with the target of 10%, these data highlight how difficult weight loss can be. Real‐world clinical outcomes amongst less motivated patients outside of a trial context is likely worse. Secondary analysis of the EAST‐AFNET 4 trial also showed that obesity did not change the effect of early rhythm control according to BMI subgroup [31]. Once again, arrhythmia recurrence outcomes in PFA amongst patients with obesity are lacking and our data provide reassurance both with regard to safety but also clinical outcomes with no difference in subsequent cardioversions, arrhythmia‐related hospitalizations or redo‐ablation procedures at short term follow‐up. No blanking period was applied in this study as we have sought to describe real‐world outcomes of patients in terms of symptomatic recurrences, cardioversions and hospitalizations. Growing data also suggest that early recurrences predict late recurrences even in the context of PFA [32]. Clinical judgment must still be exercised as to when to consider an arrhythmic recurrence a failure of procedure and time to consideration of any repeat procedure.

We acknowledge several limitations in this study. First, it is a retrospective single‐center analysis of patients undergoing PFA and remains susceptible to bias. Second, evaluation of arrhythmia recurrence was available in 62% of patients due to many patients being referred from external centers. Arrhythmia surveillance was by routine electrocardiogram at clinic review or triggered by symptoms. Routine systematic Holter monitoring was not performed post ablation and thus asymptomatic recurrences may be under‐reported. Third, follow‐up was short term at approximately 6 months. However, the documented last follow‐up was at the time of first arrhythmia recurrence which would reduce the apparent overall follow‐up period in patients with arrhythmia recurrence. Fourth, there is possibility of type II error for the detection of rarer complications and larger datasets are needed to confirm our findings. Fifth, while we sought to adjust for potential confounders on multivariate analysis regarding procedural complications and clinical outcomes, these statistical adjustments may not fully account for baseline imbalances particularly with respect to sex and associated comorbidities. Finally, all procedures were performed using a pentaspline PFA catheter and our findings may not extrapolate to other PFA device platforms.

## 5 Conclusion

Patients with obesity referred for PFA were more likely female with higher prevalence of cardiovascular comorbidities and persistent AF. Obese patients have no signal of increased risk with regards to procedural and clinical outcomes compared with nonobese patients undergoing PFA. While weight management should be encouraged, these data provide reassurance that PFA can be performed safely in patients with obesity and longer term follow‐up and clinical trials are needed.

## Funding

The authors have nothing to report.

## Ethics Statement

The study protocol, all amendments, and the informed consent form were approved by the Monash Health Human Research Ethics Committee.

## Conflicts of Interest

E.K. reports serving on the medical advisory boards for Medtronic, Boston‐Scientific, and Biotronik. Other authors declare no conflicts of interest.

## Data Availability

The data that support the findings of this study are available from the corresponding author upon reasonable request.
